# Impact of RUNX2 on drug-resistant human pancreatic cancer cells with *p53* mutations

**DOI:** 10.1186/s12885-018-4217-9

**Published:** 2018-03-20

**Authors:** Toshinori Ozaki, Meng Yu, Danjing Yin, Dan Sun, Yuyan Zhu, Youquan Bu, Meixiang Sang

**Affiliations:** 1 Laboratory of DNA Damage Signaling, Chiba Cancer Center Research Institute, Chiba, 260-8717 Japan; 2 Department of Laboratory Animal of China Medical University, Shenyang, 110001 People’s Republic of China; 3 Research Center, Fourth Hospital of Hebei Medical University, Shijiazhuang, Hebei 050017 People’s Republic of China; 4 Department of Urology, First Hospital of China Medical University, Shenyang, 110001 People’s Republic of China; 5 Department of Biochemistry and Molecular Biology, Chongqing Medical University, Chongqing, 400016 People’s Republic of China

**Keywords:** Gemcitabine, Mutant p53, p53 family, RUNX2

## Abstract

**Background:**

Despite the remarkable advances in the early diagnosis and treatment, overall 5-year survival rate of patients with pancreatic cancer is less than 10%. Gemcitabine (GEM), a cytidine nucleoside analogue and ribonucleotide reductase inhibitor, is a primary option for patients with advanced pancreatic cancer; however, its clinical efficacy is extremely limited. This unfavorable clinical outcome of pancreatic cancer patients is at least in part attributable to their poor response to anti-cancer drugs such as GEM. Thus, it is urgent to understand the precise molecular basis behind the drug-resistant property of pancreatic cancer and also to develop a novel strategy to overcome this deadly disease.

**Review:**

Accumulating evidence strongly suggests that *p53* mutations contribute to the acquisition and/or maintenance of drug-resistant property of pancreatic cancer. Indeed, certain p53 mutants render pancreatic cancer cells much more resistant to GEM, implying that *p53* mutation is one of the critical determinants of GEM sensitivity. Intriguingly, runt-related transcription factor 2 (RUNX2) is expressed at higher level in numerous human cancers such as pancreatic cancer and osteosarcoma, indicating that, in addition to its pro-osteogenic role, RUNX2 has a pro-oncogenic potential. Moreover, a growing body of evidence implies that a variety of miRNAs suppress malignant phenotypes of pancreatic cancer cells including drug resistance through the down-regulation of RUNX2. Recently, we have found for the first time that forced depletion of *RUNX2* significantly increases GEM sensitivity of *p53*-null as well as *p53*-mutated pancreatic cancer cells through the stimulation of p53 family TAp63/TAp73-dependent cell death pathway.

**Conclusions:**

Together, it is likely that RUNX2 is one of the promising molecular targets for the treatment of the patients with pancreatic cancer regardless of their *p53* status. In this review article, we will discuss how to overcome the serious drug-resistant phenotype of pancreatic cancer.

## Background

Pancreatic cancer which is highly metastatic to lymph nodes, liver and the other distal sites, is one of the most lethal malignancies among human cancers with 5-year survival rate less than 10%, and its incidence is gradually increasing worldwide [1]. Although 20% of patients receive surgical resection at diagnosis, the remaining 80% of patients are identified as unresectable due to their late diagnosis [2]. Gemcitabine (GEM), a deoxycytidine analogue, has become a standard chemotherapeutic for the treatment of patients with advanced pancreatic cancer [3]. Unfortunately, its clinical efficacy is extremely limited and the extensive efforts to develop the combination regimes with GEM have resulted in only a minor improvement over the conventional therapies [4]. Therefore, it is urgent to develop novel treatment options against pancreatic cancer, and also to understand the precise molecular mechanisms how pancreatic cancer cells could acquire and maintain GEM-resistant phenotype.

As mentioned above, GEM resistance is a critical issue to be adequately addressed for the better treatment of pancreatic cancer patients. Accumulating evidence strongly suggests that the alterations within *KRAS*, *p53*, *CDKN2A* and *SMAD4* are frequently detected in pancreatic cancer tissues, and contribute to the genesis and/or maintenance of their advanced phenotypes including GEM resistance [5]. Among these genetic aberrations, *p53* mutations (around 75%) appear in the later stages of pancreatic cancer genesis and development [6, 7]. Since p53, which monitors and ensures the genomic integrity, is an essential molecular barrier against carcinogenesis [8, 9], it is possible that loss of function mutation of *p53* leads to the accumulation of genetic damage within pancreatic cancer cells, and thus they might acquire GEM-resistant property as well as metastatic potential.

RUNX2 (also called Osf2/Cbfa1, AML-3 or Pebp2αA), a member of RUNX (runt-related transcription factor) family, has been shown to be one of the major determinants of osteoblast differentiation and bone formation [10]. As expected, RUNX2 transactivates number of pro-osteogenic target genes such as collagen type I, bone alkaline phosphatase, osteopontin and osteocalcin [11]. In addition to its pro-osteogenic role, a growing body of evidence strongly suggests that RUNX2 plays a vital role in tumor initiation, progression, invasion and metastasis. From the clinical point of view, the elevated expression level of RUNX2 has been shown to correlate to poor prognosis of patients with pancreatic cancer or with thyroid cancer [12, 13]. In support of these observations, it has been described that RUNX2 regulates numerous genes implicated in carcinogenesis including *MMP9* (matrixmetalloproteinase-9), *MMP13* (matrixmetalloproteinase-13)*, VEGF* (vascular endothelial growth factor) and *survivin* [14–16]. Furthermore, Pratap et al. found that RUNX2 promotes invasion of bone metastatic cancer cells through the induction of MMP9, and also stimulates the early events of breast cancer progression [17, 18]. Recently, we have described for the first time that siRNA-mediated knockdown of *RUNX2* increases adriamycin (ADR) sensitivity of *p53*-wild-type osteosarcoma cells through the activation of p53 family-dependent cell death pathway [19, 20]. Our subsequent studies revealed that depletion of *RUNX2* improves GEM sensitivity of pancreatic cancer cells regardless of their *p53* status [21–23].

In this review article, we provide a brief overview of the molecular basis behind drug-resistant phenotype of pancreatic cancer cells, and also describe p53 family-dependent cell death pathway in response to DNA damage. Subsequently, we summarize the current understanding of oncogenic potential of RUNX2 and possible involvement of RUNX2 and various miRNAs in pancreatic cancer. Lastly, we discuss how to overcome the serious drug-resistant phenotype of pancreatic cancer.

## Main text

### Pancreatic cancer

Pancreatic cancer is ranked as the fourth leading cause of cancer-related death in the world (both in industrial countries as well as nonindustrial ones), and is known to exhibit the worst prognosis among cancers (5-year survival rate is less than 10%) [24, 25]. Its mortality rate is nearly equal to its incidence. Up to 80% of pancreatic cancer deaths take place within the first year of diagnosis. Although surgical resection is the only potentially curative approach against pancreatic cancer, greater than 80% of cases are judged as unresectable at the time of diagnosis due to its locally advanced property and/or metastasis. A highly invasive and metastatic nature of the advanced pancreatic cancer is often responsible for its extremely poor clinical outcome. Therefore, it is urgently required to identify the reliable diagnostic and/or prognostic markers. These biomarkers could be helpful to the accurate detection of pancreatic cancer in the early stage, and the prediction of its biological behavior.

Because of the low chance of successful surgery, chemotherapy is the most common approach to extend the survival time of pancreatic cancer patients. For advanced pancreatic cancer patients, a deoxycytidine analogue termed gemcitabine (GEM) (2′,2′-difluorodeoxycytidine) has been considered to be the first choice as a front-line chemotherapy based on the results of the Phase III trial [26, 27]. The cytotoxicity of GEM relies on its ability to promote cancer cell death. Similar to the related nucleoside analog termed cytarabine (Ara-C) [28], GEM is taken up within pancreatic cancer cells through equilibrative nucleoside transporter-1 (hENT1) [29], and subjected to deoxycytidine kinase (dCK)-mediated phosphorylation to become an active form (dFdCTP). dFdCTP is then incorporated at the end of the elongating DNA strand, terminates DNA replication process, and thereby inducing DNA fragmentation (cancer cell death) [30]. In addition to the advanced pancreatic cancer, GEM is also utilized to treat patients with the other serious diseases such as non-small-lung cell, breast, bladder, gastric and ovarian cancers. However, the response rate of GEM monotherapy is low and the improvement of 5-year survival rate is unsatisfied (less than 6 months) [31]. The efficacy of GEM is less than 20% of treated patients, and almost all the patients eventually become resistant to GEM [31].

Subsequently, the extensive studies to improve the unfavorable clinical outcome of the advanced pancreatic cancer patients by GEM-based combination therapies with the other anti-cancer drugs including fluorouracil, irinotecan, pemetrexed, oxaliplatin, exatecan, cisplatin and capecitabine, have been performed. Unfortunately, most of these combination therapies have failed to obtain much better results than GEM monotherapy [32–34]. Recently, it has been shown that the combination of GEM with erlotinib (Erlo) or GEM plus nab-paclitaxelm (nab-PTX) has marginal benefits in survival rate of patients with the advanced pancreatic cancer [35, 36]. In support of these observations, it is well known that pancreatic cancer is intrinsically resistant to GEM or acquires GEM resistance. To override GEM-resistant property of pancreatic cancer and improve clinical outcome of the patients, it is critical to understand the precise molecular mechanisms behind its GEM-resistance, and also to develop more efficient GEM-based treatment options for the patients.

### Molecular basis underlying GEM-resistance of pancreatic cancer

As mentioned above, drug resistance is a hallmark of pancreatic cancer, and the poor clinical outcome of the patients is partly due to its drug-resistant phenotype. To date, various hypotheses explaining its drug resistance have been postulated. Firstly, it has been generally accepted that its drug resistance is generated by the aberrant overexpression of P-glycoprotein as well as the other transporters, and thereby reducing the accumulation of anti-cancer drugs inside cancer cells [37, 38]. For example, multidrug-resistance 1 (MDR1/ ABCB1/P-glycoprotein) is a transmembrane glycoprotein of 170 kDa and acts as an ATP-dependent drug-efflux pump. O'Driscoll et al. reported that MDR1 is expressed in the majority of pancreatic cancer tissues, indicative of its important contribution to their drug resistance [39]. Consistent with these observations, Song et al. demonstrated that depletion of *MDR1* sensitizes GEM-resistant pancreatic cancer Panc-1 cells to GEM [40]. Intriguingly, Zhang et al*.* found that Ser/Thr kinase PLK1 is expressed at higher level in pancreatic cancer tissues as compared to normal pancreatic ones, and a potent PLK1 inhibitor DMTC acts synergistically with GEM [41]. Recently, Li et al*.* demonstrated that PLK1 inhibitor GSK461364A enhances the efficacy of GEM [42]. As described [40], PLK1 augmented c-fos-mediated induction of MDR1, suggesting that PLK1 reduces GEM sensitivity of pancreatic cancer cells through up-regulation of MDR1. Another transporter responsible for drug resistance is MDR-related protein MRP1/2 (ABCC1/2). Lee et al. showed that MRP1 and MRP2 are expressed in 84 and 91% of pancreatic cancer cases [43]. Nath et al. found that a significantly higher expression level of *MRP1* is closely associated with GEM-resistant phenotype of pancreatic cancer cells [44].

Since the diffusion of hydrophilic GEM through the plasma membrane lipid bilayer is very slow, the efficient cellular uptake of GEM requires the specialized membrane nucleoside transporter protein(s) [45]. Among the nucleoside transporters, it has been shown that human equilibrative nucleoside transporter 1 (hENT1) plays a major role to facilitate the intracellular uptake of GEM across the plasma membrane [46]. Indeed, *ENT1*-deficient cells were highly resistant to GEM [45], and forced expression of hENT1 enhanced GEM response of pancreatic cancer cells [47]. In accordance with these results, hENT-positive pancreatic cancer patients had a prolonged survival rate after GEM treatment relative to the patients without detectable hENT [48]. Similarly, Nordh et al*.* described that the expression level of hENT is a reliable predictive indicator for pancreatic cancer patients treated with GEM [49]. Therefore, it is likely that the defect(s) in hENT-mediated intracellular uptake of GEM renders pancreatic cancer cells resistant to GEM.

Meanwhile, it has been well documented that tumor suppressor *p53* is frequently mutated in pancreatic cancer tissues (around 75%) [50], indicating that mutant p53 contributes to the development of GEM-resistant nature of pancreatic cancer. Notably, Florini et al. detected GEM-mediated stabilization of mutant p53 at protein level in pancreatic cancer cells [51]. We have also observed the similar phenomenon [22]. In the next chapter, we would like to describe p53 and its family members.

### p53-dependent cell death pathway

**Fig. 1:**
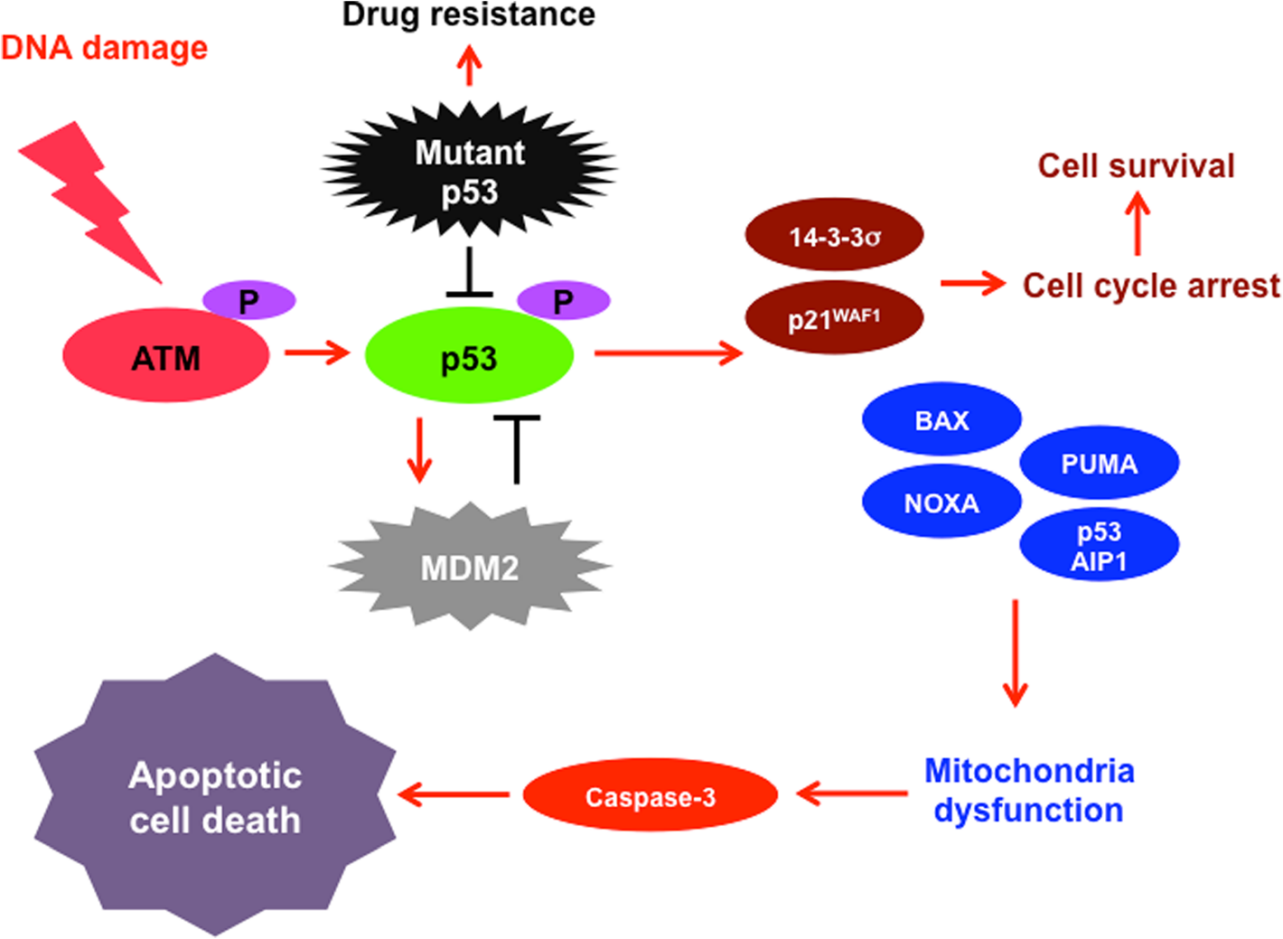
p53-dependent cell death pathway. Upon DNA damage, p53 becomes activated through ATM-mediated phosphorylation, and transactivates pro-arrest *p21*^*WAF1*^ and/or 14-3-3σ as well as pro-apoptotic *BAX*, *NOXA*, *PUMA* and/or *p53AIP1*. The accumulation of these small mitochondrial proteins promotes mitochondria dysfunction followed by caspase-3 activation, and then cells undergo cell death

*p53* is one of the well-studied tumor suppressor genes. Since p53 is able to protect cells from serious DNA damage and thus prevent carcinogenesis, p53 is called “guardian of the genome” [52]. Under the normal conditions, p53 is kept at an extremely low level. Upon cellular stresses such as DNA damage, oncogene activation, hypoxia and telomere shortening, p53 becomes stabilized at protein level, activated through the sequential post-translational modifications and then triggers a cascade of the molecular events which determines cell fate such as cell cycle arrest, cellular senescence and/or cell death [53]. These chemical modifications including phosphorylation and acetylation, which attenuate MDM2 (murine double minute 2 homolog)-mediated degradation of p53, extend the half-life of p53, and thereby increasing the intracellular p53 level. MDM2 with an E3 ubiquitin protein ligase enzymatic activity, catalyzes poly-ubiquitination of p53 and facilitates its rapid degradation through proteasome [54, 55]. Once activated, p53 functions as a sequence-specific transcription factor to transactivate a large number of its downstream target genes implicated in the regulation of the above-mentioned cellular processes. For example, p21^WAF1^ and 14-3-3σ are involved in the induction of p53-dependent G1/S and G2/M cell cycle arrest (cell survival), respectively. While, p53-mediated transcriptional activation of BAX, PUMA, NOXA and/or p53AIP1 participates in the proper cell death response. Therefore, p53 stands at the crossroad between cell survival and death, which might rely on the intensity of stress signal and/or extent of cellular damage (Fig. 1).

*p53* is frequently mutated in around 50% of human cancers [56]. The majority of mutations occur within its central core sequence-specific DNA-binding domain with 6 hot spots at codons such as R175, G245, R248, R249, R273 and R282, and result in the production of conformationally aberrant p53 proteins (mutant p53). *p53* hot spot mutations account for 30% of all reported ones. These observations indicate that mutant p53 lacks the sequence-specific transactivation capability. Since pro-apoptotic function of wild-type p53 is tightly linked to its sequence-specific transcriptional activity, mutant p53 fails to suppress tumor initiation as well as progression. Of note, most common *p53* mutations not only impair its tumor-suppressor function (loss of function) but also confer novel pro-oncogenic potential on p53 (gain of function), which markedly enhances tumor progression and drug resistance [57]. Knock-in mice expressing mutant p53 (R175H and R273H) displayed an accelerated tumor growth, which was more invasive and metastatic relative to that of *p53*-deficient mice [58]. In addition to R175H and R273H mutants, Hanel et al. found that R248 mutant mice show gain of function in carcinogenesis [59]. These findings strongly suggest that at least certain p53 mutants exhibit gain of function. In support of this notion, it has been demonstrated that mutant p53 has an ability to transactivate various pro-oncogenic genes such as *MYC*, *PDGFR*, *HGFR*, *EGFR* and *MDR1* [60]. Recently, Zhao et al. described that Pontin with an ATPase activity interacts with mutant p53 and facilitates its transcriptional activity [61]. Intriguingly, overexpression of Pontin is detectable in a variety of cancer tissues, and strongly associated with poor prognosis of the patients [62]. In addition to Pontin, PML and Pin1 have been shown to enhance the transcriptional capability of mutant p53 through the direct interaction [63, 64].

In a sharp contrast to wild-type p53 with an extremely short half-life (around 20 min), mutant p53, which escapes from ubiquitin/proteasome-dependent degradation pathway mediated by MDM2, has an extended half-life. It has been shown that molecular chaperone Hsp90 is associated with mutant p53, prevents its degradation, and thus facilitates its accumulation in cancer cells [65]. The strong immunohistochemical positivity for p53 has been employed as a diagnostic indicator for the presence of a *p53* mutation [57]. Since the vast majority of *p53* mutations occur within its central core sequence-specific DNA-binding domain, mutant p53 retains an intact COOH-terminal oligomerization domain. Therefore, a large amount of mutant p53 forms a hetero-oligomer with wild-type p53 *via* its oligomerization domain through which mutant p53 displays a dominant-negative behavior against wild-type p53 [66]. This hetero-oligomerization diminishes the tumor-suppressive function of wild-type p53. Emerging evidence suggests that *p53* status of cancer cells is closely associated with their sensitivity to anti-cancer drugs [67–69]. Together, the gain of function (GOF) and/or loss of function (LOF) mutations of *p53* might be one of the possible molecular mechanisms of the serious drug resistance of cancer cells. Considering that one half of cancer cells express wild-type p53 but not mutant p53, the presence of *p53* mutation-independent mechanisms, which could disrupt p53-dependent cell death pathway, should also be kept in mind.

### MDM2, a negative regulator of p53

As described above, around half of cancer patients carry wild-type *p53*, raising a question why the patients harboring wild-type *p53* sometimes do not respond to the standard chemotherapy. A number of evidence indicates that, apart from mutant p53, the other cellular factors might directly prohibit wild-type p53 and/or disrupt the upstream or downstream p53-dependent cell death pathway. Among them, MDM2 is one of the major negative regulators of p53. MDM2 deficiency promoted p53-dependent cell death in a variety of cells [70]. *p53* is infrequently mutated in glioblastoma; however, wild-type p53 remains dysfunctional due to the overexpression of MDM2 [71]. In addition to the stimulation of proteasomal degradation of p53, MDM2 binds to NH_2_-terminal transactivation domain I (TD1) of p53, strongly prohibits it from serving as a transcriptional activator, and thereby attenuates p53-mediated cell death in response to DNA damage. Alternatively, MDM2 interacts with p53 and drives its sequestration in the cytoplasm. Subcellular localization of p53 is regulated through its ubiquitination status. It has been shown that MDM2-mediated monoubiquitination of p53 induces its nuclear export and polyubiquitination facilitates its proteasomal degradation [72].

Since *MDM2* is one of the downstream target genes of p53, its expression is tightly regulated in a p53-dependent manner. Thus, p53 modulates the intracellular expression level of its own negative regulator MDM2 *via* a feedback loop [73]. As a result of this regulatory system, the amounts of p53 and MDM2 are maintained at extremely low level under the healthy conditions. In contrast to normal cells, *MDM2* gene is sometimes amplified and/or aberrantly overexpressed in number of cancers including pancreatic cancer [74, 75]. The overall frequency of *MDM2* gene amplification in human cancers is around 7%; however, cancer tissues such as osteosarcoma (16%), soft tissue tumors (31%), hepatocellular carcinoma (44%) and Hodgkin disease (67%) have the higher frequencies of *MDM2* gene amplification [76]. It is worth noting that the elevated level of *MDM2* expression is significantly associated with poor clinical outcome of the patients with pancreatic cancer [77, 78]. In a good agreement with these observations, the abnormal overexpression of MDM2 caused by its gene amplification and/or transcriptional activation mediated by p53, disrupts the balance between the intracellular amounts of p53 and MDM2, and then promotes tumor development [79]. Wade et al. described that a 2-fold increase in MDM2 expression level is enough to prohibit p53 activation [80]. As expected, shRNA-mediated knockdown of *MDM2* suppressed proliferation rate and tumor growth potential of highly metastatic pancreatic cancer cells [81]. Under their experimental conditions, depletion of *MDM2* caused an up- and down-regulation of *p53* and *MMP9*, respectively.

Consistent with those results, Kondo et al. have described that MDM2 plays a vital role in the development of resistance to CDDP (cisplatin) in human glioblastoma cells [82]. Suzuki et al. demonstrated that forced expression of MDM2 overrides wild-type p53 and confers ADR (adriamycin) resistance on breast cancer cells [83]. Meijer et al. reported that a small chemical compound termed Nutlin-3, which binds to MDM2 and inhibits its interaction with p53, preferentially enhances drug-sensitivity of wild-type *p53*-expressing ovarian cancer cells through the accumulation of wild-type p53 [84]. Collectively, MDM2-mediated dysfunction of p53 is one of the primary molecular mechanisms underlying *p53* mutation-independent acquisition of chemo-resistance.

### p53 family

Following the identification of p53, two independent p53-related nuclear transcription factors such as p73 and p63 have been discovered (p53 family members) [85, 86]. These p53 family members have a similar exon/intron organization and display a remarkable amino acid sequence similarity especially in their central DNA-binding domains (around 63%). Subsequent studies revealed that both *p73* and *p63* genes encode multiple variants, which are basically divided into two groups such as transcription-competent TA (TAp73 and TAp63) and transcription-deficient ΔN (ΔNp73 and ΔNp63) isoforms. The alternative splicing events produce various TA isoforms with the distinct COOH-terminal portions as well as the different tranactivation potential. While, the alternative promoter usage gives rise to NH_2_-terminaly truncated ΔN isoforms, which lack the first TA (transactivation) domain. As expected from their differential transactivation potentials, TA and ΔN isoforms have their own physiological functions. As described [87, 88], *TAp73*- or *TAp63*-null mice developed spontaneous tumors, indicating that, like p53, TAp73 and TAp63 act as tumor suppressors. On the other hand, *ΔNp73*- or *ΔNp63*-deficient mice displayed complex developmental defects in the nervous system or in the epidermis and limbs, respectively [89, 90].

Like p53, TAp73/TAp63 are induced following genotoxic insults, recruited onto p53-responsive elements within the promoter regions of the overlapping set of p53-target pro-apoptotic genes including *BAX*, *PUMA*, *NOXA* and/or *p53AIP1*, and then efficiently promote cell death [91]. The expression of TAp73 is regulated at both mRNA and protein levels. Lissy et al. revealed that *TAp73* is transactivated by E2F-1 in response to cell death stimulus [91]. Basically similar results were also reported from several independent groups, suggesting that E2F-1 triggers cell death through the activation of TAp73-dependent cell death pathway [92–94]. Alternatively, several lines of evidence suggest that Sp1 (specificity protein 1) and Nrf-2 (nuclear factor erythroid 2-related factor 2) stimulate *TAp73* transcription [95, 96]. By contrast, Fontemaggi et al. found that *TAp73* expression is down-regulated by the transcriptional repressor ZEB-1 during differentiation [97]. While, Rossi et al. found that HECT domain-containing Nedd4-like E3 ubiquitin ligase Itch binds to TAp73/TAp63 and promotes their proteolytic degradation through proteasome [98, 99]. According to their results, the expression level of Itch was significantly reduced in response to DNA damage caused by ADR, VP16 or ADR, implying that DNA damage-mediated reduction of Itch contributes at least in part to the increased stability of TAp73/TAp63. Consistent with these observations, Hansen et al. described that silencing of *Itch* increases drug sensitivity of cancer cells even in the absence of functional p53 [100]. Notably, it has been shown that TAp73/TAp63 are required for p53-dependent cell death in response to DNA damage, whereas TAp73/TAp63 induce DNA damage-mediated cell death without functional p53 [101]. Since *p73*/*p63* are rarely mutated in human cancer tissues [102], TAp73/TAp63 are expressed as the functional wild-type forms, raising a possibility that, instead of wild-type p53, TAp73/TAp63 might trigger DNA damage-mediated cell death in *p53*-null and/or *p53*-mutated cancer cells.

**Fig. 2:**
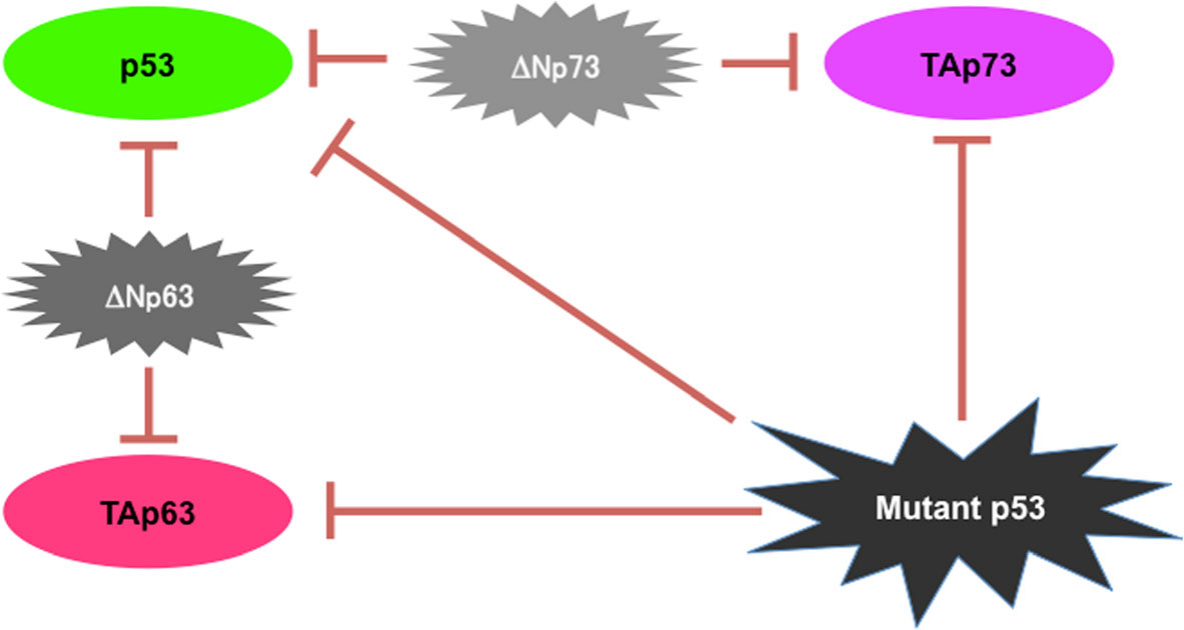
Functional interplay among p53 family members. Mutant p53 inhibits pro-apoptotic wild-type p53, TAp73 and TAp63 through the direct interaction, and thus contributes to the acquisition and/or maintenance of the serious drug-resistant phenotype of malignant tumors

Unlike TAp73/TAp63, ΔNp73/ΔNp63 lack the acidic NH_2_-terminal transactivation domain. As expected, ΔNp73/ΔNp63 fail to specifically transactivate p53-target gene promoters, although they are capable to bind to p53-responsve elements within them. Intriguingly, it has been described that ΔNp73/ΔNp63 exert their own transcriptional activities, which is dependent on two additional transactivation domains located between their COOH-terminal oligomerization domain and SAM domain, and close to the Pro-rich domain [103]. Therefore, ΔNp73/ΔNp63 have an ability to activate their own downstream target gene promoters [104]. For example, Wu et al. found that ΔNp63 activates pro-metastatic *Hsp70* gene transcription [105]. Soldevilla et al. reported that *ABCB1* and *HMGB1* are the putative downstream target genes of ΔNp73 [106]. In addition to the transactivation of pro-oncogenic genes, ΔNp73/ΔNp63 act as the dominant-negative inhibitors against TAp73/TAp63 and wild-type p53 through the formation of the inactive complexes with them or the competition for promoter binding sites [107]. Indeed, ΔNp73 is overexpressed in a variety of cancers including lung, breast, brain, thymus, colon, ovary, skin and prostate cancers [108, 109], and this up-regulation of ΔNp73 has been shown to tightly link to poor prognosis of cancer patients [110]. Leung et al. demonstrated that ΔNp73 contributes to the development of CDDP resistance of ovarian cancer cells through the activation of pro-oncogenic AKT signaling pathway [111]. For ΔNp63, its overexpression was detectable in head and neck, lung, esophagus, bladder, liver and tongue cancers [112, 113], and the increased expression of ΔNp63 has been considered to be unfavorable clinical indicator of patients with melanoma [114]. Rocco et al*.* found that ΔNp63 attenuates TAp73-dependent cell death pathway, and acts as the major determinant of CDDP sensitivity of head and neck cancer [115]. Together, it is likely that, although mutant p53 and ΔNp73/ΔNp63 have a strong dominant-negative potential against TAp73/TAp63, the response to anti-cancer drugs of cancer cells lacking wild-type p53 might be determined at least in part by functional TAp73/TAp63 (Fig. 2). With these in mind, we have to discuss especially how to override the negative effect of mutant p53 on TAp73/TAp63.

### Pro-oncogenic RUNX2

Runt-domain containing transcription factor 2 (also called Osf2/Cbfa1, AML-3 or Pebp2αA) is a member of RUNX family, which is identified by an evolutionary conserved DNA-binding/protein-protein interaction domain called runt-homology domain. In contrast to the other RUNX family members such as RUNX1 and RUNX3 whose mutations are tightly linked to the promotion of leukemia and gastric cancer, respectively [116, 117], the initial studies strongly suggest that RUNX2 acts as a master regulator of osteoblast differentiation and bone development. In support of this notion, *RUNX2*-deficient mice died shortly after birth due to a complete lack of bone formation with arrest of osteoblast differentiation [118, 119]. During bone development, RUNX2 facilitates the differentiation of mesenchymal stem cells into the osteoblast lineage [120]. As expected, RUNX2 transactivates its downstream target gene promoters implicated in these cellular and developmental processes including collagen type I, bone alkaline phosphatase, osteopontin and osteocalcin. Of note, Liu et al*.* demonstrated that RUNX2 is a prerequisite for the early stage of osteoblast differentiation, whereas its overexpression attenuates the subsequent osteoblast maturation [121]. Thus, it is indicative that *RUNX2* is under the strict transcriptional and/or post-translational regulation during the cellular differentiation as well as development.

Recently, the possible involvement of RUNX2 in tumor initiation/progression has been increasingly recognized depending on the cellular context. In addition to osteogenesis-related genes, RUNX2 has an ability to transactivate its downstream target genes involved in tumor progression, invasion and metastasis such as *MMP9*, *MMP13*, *VEGF*, *survivin*, *IL-8* and *TGFβR* [14–16, 122–124]. The extensive expression studies demonstrated that the expression level of RUNX2 is aberrantly elevated in numerous cancer tissues as compared to their corresponding normal ones including pancreatic cancer, breast cancer, prostate cancer and osteosarcoma [125]. Of note, overexpression of RUNX2 has been shown to cause a poor response to chemotherapy of certain cancer cells. For example, *RUNX2* gene is sometimes amplified, overexpressed in osteosarcomas and thus has been employed as a reliable marker for the estimation of their chemo-resistance [126, 127]. Roos et al. have revealed that loss of RUNX2 expression increases ADR sensitivity of osteosarcoma cells [128]. Similarly, RUNX2 was aberrantly overexpressed in breast cancer cells and promoted their progression [17, 18, 129]. Consistent with these observations, El-Gendi and Mostafa have described that the expression level of RUNX2 is a potential prognostic indicator for breast cancer [130]. As expected, targeting of *RUNX2* suppressed breast cancer progression and bone metastasis [131]. Additionally, it has been shown that the abnormally higher RUNX2 expression level in prostate cancer tissues is positively associated with their stage and aggressiveness [132]. For pancreatic cancer, Kayed et al*.* found that the increased expression level of *RUNX2* correlates to unfavorable prognosis of the patients with this serious disease [12]. Given those findings, it is suggestive that RUNX2 is a potential diagnostic marker and/or therapeutic target of the advanced tumors such as pancreatic cancer.

Growth of solid tumors including pancreatic cancer often depends on angiogenesis with the continuous blood vessel formation, and thus the inhibition of tumor angiogenesis is a potential strategy for cancer treatment [133]. Indeed, numerous studies have shown that the attenuation of tumor angiogenesis prohibits pancreatic cancer growth and metastasis [134–136]. Among RUNX2-target gene products as described above, VEGF is the most potent angiogenic cytokine [137]. Through the binding to its receptors (VEGFR-1 and VEGFR-2) whose expression is predominantly restricted to endothelial cells in blood vessels, VEGF triggers a variety of downstream survival and migration pathways, and then induces angiogenesis. For transcriptional regulation of *VEGF*, a large body of evidence suggests that *VEGF* is transcriptionally activated by hypoxia-inducible factor (HIF). Hypoxia is a major stimulator of angiogenesis. HIF is a heterodimeric transcription factor composed of an oxygen-sensitive alpha subunit (HIF-1α or HIF-2α) and a constitutively expressed beta subunit (HIF-1β). Under normoxic conditions, HIF-1α and HIF-2α are subjected to a rapid hydroxylation at their proline residues, followed by proteasomal degradation mediated by an E3 ubiquitin ligase VHL (von Hippel-Lindau). By contrast, HIF-1α as well as HIF-2α is stabilized at protein level due to the strong inhibition of its hydroxylation and ubiquitination under hypoxic conditions, forms an active HIF transcriptional complex with its binding partner HIF-1β in cell nucleus and regulates its downstream target gene expression. Since HIF-1β is not affected by oxygen concentration and is expressed in excess, the protein levels of HIF-1α and HIF-2α are responsible for HIF transcriptional activity [138, 139]. As expected from those observations, HIF-1α was highly expressed in several solid tumors, and forced suppression of HIF-1α impaired tumor angiogenesis, growth and metastasis [140, 141].

In addition to HIF transcription complex, Zelzer et al. demonstrated for the first time that RUNX2 directly binds to *VEGF* promoter region, and stimulates its transcription [142]. According to their results, targeting *RUNX2* resulted in a significant loss of VEGF expression in mice, and overexpression of RUNX2 in cultured murine fibroblasts caused an obvious increase in *VEGF* mRNA level. Alternatively, Lee et al. found that RUNX2 has an ability to increase the protein stability and transcriptional activity of HIF-1α by competing with VHL to block its ubiquitination [143]. From their results, ectopic expression of RUNX2 promoted the nuclear access of HIF-1α, elevated the secretion of VEGF, and augmented the *in vitro* and *in vivo* angiogenesis, indicative that RUNX2 acts as a potent inducer of angiogenesis through the enhancement of HIF-1α-dependent transactivation of *VEGF*. Intriguingly, Rhaman et al have reported that VEGF enhances the protein stability of RUNX2 by blocking its degradation, indicating the presence of a positive feedback loop between VEGF and RUNX2, which might synergistically modulate angiogenesis [144]. In a sharp contrast to RUNX2, it has been shown that RUNX3 is recruited onto the putative RUNX3-binding sites of *VEGF* promoter, trans-represses its transcription, and suppresses gastric cancer angiogenesis [145]. Peng et al. described that RUNX1 physically interacts with HIF-1α and prohibits its transcriptional activity [146]. Under their experimental conditions, forced expression of RUNX1 resulted in a marked decrease in *VEGF* mRNA level. These observations raise a possibility that RUNX family members might differentially regulate angiogenesis. Since, among RUNX family proteins, RUNX2 strongly stimulates VEGF-dependent angiogenesis, RUNX2 might be an attractive molecular target for therapies, which seek to repress malignant progression of solid tumors such as pancreatic cancer.

### Functional interplay between p53 family and RUNX2

Previously, we have found that tumor-suppressive RUNX3 enhances pro-apoptotic activity of p53 in osteosarcoma-derived U2OS cells exposed to ADR through the stimulation of ATM-dependent phosphorylation of p53 at Ser-15 [147]. Subsequent studies revealed that another RUNX family member RUNX1 facilitates p300-mediated acetylation of p53 at Lys-373/382 and thus augments p53-induced cell death of U2OS cells in response to ADR [148]. We then asked whether there could exist a functional interaction between p53 and the remaining RUNX family member RUNX2. In a sharp contrast to RUNX1 and RUNX3, we have found for the first time that RUNX2 strongly prohibits p53/TAp73-mediated cell death of U2OS cells following ADR exposure. Based on our prior results, RUNX2 was associated with histone deacetylase HDAC6 as well as p53 and impaired its transcriptional and pro-apoptotic activities. In addition to p53, RUNX2 repressed *TAp73* transcription and also bound to TAp73 to diminish its pro-apoptotic activity. These observations were in accordance with the recent findings of Roos et al. showing that shRNA-mediated knockdown of *RUNX2* increases ADR sensitivity of osteosarcoma cells [128].

**Fig. 3:**
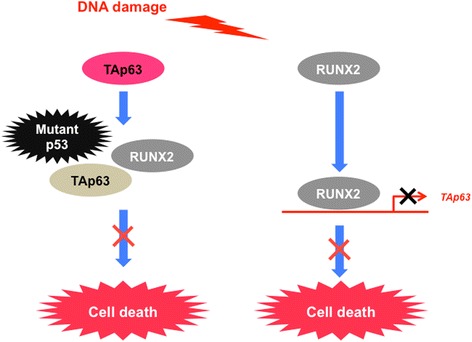
RUNX2 prohibits pro-apoptotic TAp63 in *p53*-mutated pancreatic cancer cells. RUNX2 collaborates with mutant p53 to inhibitpro-apoptotic TAp63 in pancreatic cancer Panc-1 cells exposed to GEM. In addition to the direct interaction, RUNX2 trans-represses *TAp63* transcription

As described above, U2OS cells bearing wild-type *p53* undergo cell death following DNA damage primarily in a p53-dependent manner. Therefore, it is of interest to ask whether RUNX2 could be also involved in poor drug response of *p53*-null or *p53*-mutated cancer cells. To this end, we have employed pancreatic cancer cells in which *p53* is frequently mutated. Firstly, Sugimoto et al. revealed that depletion of *RUNX2* improves GEM sensitivity of *p53*-negative pancreatic cancer AsPC-1 cells through the stimulation of TAp63-dependent cell death pathway [21]. Secondary, Nakamura et al. demonstrated that GEM sensitivity of *p53*-mutated pancreatic cancer MiaPaCa-2 cells is increased by *RUNX2* depletion-mediated up-regulation of TAp73 [22]. Recently, we have found that RUNX2 suppresses TAp63 expression and also impairs its pro-apoptotic activity in *p53*-mutated pancreatic cancer Panc-1 cells [23]. These observations imply that RUNX2 is implicated in poor response to GEM of pancreatic cancer cells lacking functional p53, and TAp73/TAp63 might potentiate GEM-induced cell death of *RUNX2*-knocked down pancreatic cancer cells instead of wild-type p53 (Fig. 3). Previously, Flores et al. described that p53 requires TAp73 and/or TAp63 for DNA damage-induced cell death, whereas TAp73 or TAp63 is capable to promote cell death in response to DNA damage without functional p53 [101]. Thus, it is possible that *RUNX2* gene silencing-mediated up-regulation of TAp63 augments GEM-induced cell death of *p53*-null AsPC-1 cells. For *p53*-mutated MiaPaCa-2 and Panc-1 cells, it has been shown that mutant p53 acts as a strong dominant-negative inhibitor against TAp73 and TAp63 [149].

The question is how *RUNX2* depletion could partially override the negative effect of mutant p53 on TAp73/TAp63. Recently, Wang et al. reported that, in contrast to *RUNX2*, low ATF3 (activating transcription factor 3) expression level is significantly associated with poor survival of prostate cancer patients, indicating that ATF3 might serve as a tumor suppressor against prostate cancer [150]. In line with these findings, *ATF3* deficiency promoted prostate cancer development in *PTEN*-knockout mice [151]. Of note, Wei et al. found that ATF3 binds to COOH-terminal portion of mutant p53 and diminishes its pro-oncogenic activity [152]. Forced expression of ATF3 sensitized mutant p53-expressing cancer cells to CDDP or VP16. Further analysis demonstrated that ATF3 disrupts the physical interaction between mutant p53 and TAp63, and thereby facilitates the reactivation of TAp63. In addition to TAp63 reactivation, ATF3 impaired pro-oncogenic activity of mutant p53 through the direct binding. Therefore, it is suggestive that *RUNX2* depletion might enhance ATF3 expression and/or activity and thereby augment TAp63-mediated cell death even in the presence of a large amount of mutant p53. However, Gokulnath et al. described that ATF3 is efficiently recruited onto *RUNX2* promoter and stimulates its transcription in bone metastatic breast cancer cells, indicating that *RUNX2* is a direct downstream target gene of ATF3 [153]. Further studies should be necessary to verify the functional significance of the interaction among RUNX2, ATF3, mutant p53 and TAp63 in the acquisition and/or maintenance of drug-resistant phenotype of pancreatic cancer cells.

### Implication of microRNA-mediated down-regulation of *RUNX2* in pancreatic cancer

MicroRNAs (miRNAs) are a family of small (20-25 nucleotides in length) and single-stranded non-coding RNAs, which bind to the 3’-untranslated region of their target mRNAs in a sequence-specific manner, and repress their expressions through mRNA degradation and/or translation inhibition [154, 155]. An individual miRNA has a capability to regulate numerous distinct mRNAs. Intriguingly, miRNAs act as either oncogenes or tumor suppressor genes, which might be dependent on their target genes. It has been described that the dysregulated expression of certain miRNAs is closely associated with proliferation rate, invasion potential and chemo-sensitivity of pancreatic cancer cells [156]. For example, Lu et al. found that miR-301a whose expression level is specifically elevated in pancreatic cancer tissues, contributes to the persistent activation of NF-κB-mediated pro-oncogenic signaling pathway [157]. By contrast, Liang et al. demonstrated that miR-33a is capable to increase the sensitivity of pancreatic cancer cells to GEM through the down-regulation of pro-oncogenic AKT/Gsk-3β/β-catenin signaling pathway [158]. Ji et al. reported that miR-34 family members have tumor-suppressive function downstream of p53, and their restoration renders *p53*-mutated pancreatic cancer cells 2-3-fold more sensitive to GEM [159].

Meanwhile, Huang et al. found the inverse relationship between the expression levels of miR-204/miR-211 and RUNX2 during adipocyte differentiation, and also demonstrated for the first time that miR-204/miR-211 bind to the 3’-untranslated region of *RUNX2*, and attenuate its expression, indicating that miR-204/miR-211 act as the negative regulators of *RUNX2* [160]. In support of their observations, Wu et al*.* described that miR-30 family members prohibit BMP-2-induced osteoblast differentiation by targeting *RUNX2* [161]. It has been shown that the additional miRNAs (miR-23a, miR-34c, miR-133a, miR-135a, miR-205 and miR-217) also attenuate osteogenesis by targeting *RUNX2* [162, 163]. In addition to adipocyte and osteoblast differentiation, Saini et al. revealed that ectopic expression of miR-203 impairs the development of metastasis originated from prostate cancer in association with the down-regulation of pro-metastatic genes such as *ZEB2*, *survivin* and *RUNX2* [164]. van der Deen et al. described that p53-mediated stimulation of miR-34c expression causes a massive decrease in RUNX2 and reduces the metastatic potential of osteosarcoma cells [165]. Considering that the aberrant overexpression of RUNX2 correlates to resistance to chemotherapy [166], it is indicative that miR-34c contributes to the improvement of chemo-sensitivity of drug-resistant osteosarcoma cells through the down-regulation of RUNX2. Moreover, Li et al. found that the lower expression level of miR-23b is associated with worse prognosis of ovarian cancer patients, and miR-23b-induced repression of RUNX2 slow downs ovarian cancer cell proliferation [167]. Recently, Taipaleenmäki et al. showed that malignant phenotypes of breast cancer cells are significantly suppressed by miR-135/miR-203-caused direct reduction of RUNX2. Together, these observations suggest that *RUNX2*-targeting miRNAs effectively suppress the progression and/or metastasis of various types of aggressive tumors including pancreatic cancer.

**Fig. 4:**
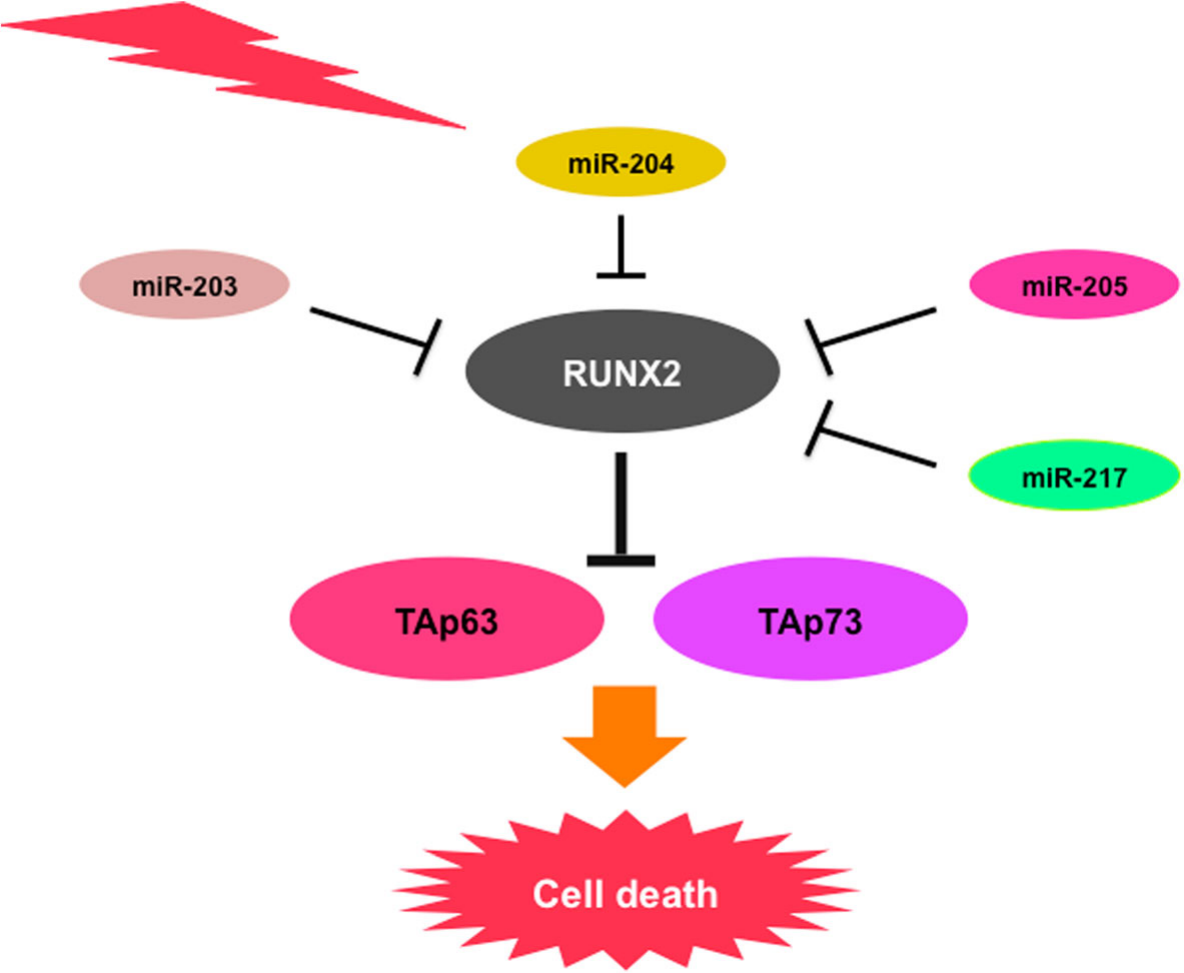
RUNX2 is a potential therapeutic target for pancreatic cancer. siRNA- and/or synthetic microRNA-mediated down-regulation of pro-oncogenic RUNX2 augments TAp73/TAp63-dependent cell death pathway, and enhances chemo-sensitivity of pancreatic cancer cells

Notably, miR-203, which prohibits prostate cancer cell metastasis, has also been shown to reduce migration/invasion capacity of pancreatic cancer cells [168]. Chen et al. reported that miR-204 is highly expressed in normal pancreatic ductal tissues relative to pancreatic cancer tissues, and promotes pancreatic cancer cell death [169]. miRNA profiling studies revealed that miR-205 is down-regulated in GEM-resistant pancreatic cancer cells and metastatic pancreatic cancer tissues [170]. Zhao et al. found that the expression level of miR-217 is significantly lower in pancreatic cancer tissues as compared to that in normal ones, and exogenous miR-217 reduces tumor growth in mouse xenograft models [171]. As mentioned above, these miRNAs such as miR-203, miR-204, miR-205 and miR-217 negatively regulate RUNX2 expression, raising a possibility that miRNA-induced down-regulation of RUNX2 contributes to the suppression of malignant properties of pancreatic cancer cells such as drug resistance (Fig. 4).

## Conclusions

Since the patients with pancreatic cancer show the worst prognosis despite the extensive therapy, it is urgent to develop a novel strategy to enable its early detection and increase its drug sensitivity. For this purpose, the precise understanding of the biology of pancreatic cancer and also molecular mechanisms how pancreatic cancer cells could acquire and maintain this serious drug-resistant phenotype should be required. An increasing body of evidence has demonstrated that RUNX2 is aberrantly overexpressed in numerous cancer tissues including pancreatic cancer relative to their corresponding normal ones, and its depletion suppresses their malignant phenotypes such as migration, invasion, metastasis and drug resistance. Consistent with these observations, we have demonstrated that siRNA-mediated knockdown of *RUNX2* improves GEM sensitivity of various pancreatic cancer cells regardless of their *p53* status. According to our results, *RUNX2* gene silencing increased GEM sensitivity of *p53*-null pancreatic cancer AsPC-1 cells [21] as well as *p53*-mutated pancreatic cancer MiaPaCa-2 and Panc-1 cells [22, 23], raising a possibility that *RUNX2* depletion improves GEM sensitivity of pancreatic cancer cells without functional p53. In a sharp contrast to *p53*, *TAp73*/*TAp63* is rarely mutated in cancer tissues. Of note, it has been shown that pro-apoptotic p53 family TAp73/TAp63 has an ability to promote DNA damage-mediated cell death in the absence of functional p53. Mutant p53 acts as a strong dominant-negative inhibitor against p53 family members; however, *RUNX2* depletion-mediated up-regulation of TAp73 and/or TAp63 resulted in an increase in GEM sensitivity of *p53*-mutated pancreatic cancer cells, which might be at least in part due to the disruption of the intracellular balance between the amounts of mutant p53 and TAp73/TAp63. Furthermore, miRNAs targeting *RUNX2* suppress malignant phenotypes of pancreatic cancer cells, suggesting that the delivery of chemically stable synthetic miRNAs to pancreatic cancer tissues is an attractive strategy to treat advanced pancreatic cancer patients. Although it is unknown whether miRNA-mediated down-regulation of RUNX2 could lead to the potentiation of TAp73/TAp63-dependent cell death pathway, *RUNX2* silencing-mediated restoration of TAp73/TAp63 and down-regulation of its pro-oncogenic downstream target genes might represent a promising approach to override the serious drug-resistant phenotype of pancreatic cancer with *p53* mutation.

## Abbreviations

ADR: Adriamycin
ATF3: Activating transcription factor 3
ATM: Ataxia telangiectasia mutated
GEM: Gemcitabine
HDAC: Histone deacetylase
hENT1: Equilibrative nucleoside transporter-1
HIF: Hypoxia-inducible factor
MDM2: Murine double minute 2
MDR1: Multidrug-resistance 1
miRNA: microRNA
MMP9: Matrixmetalloproteinase-9
MMP13: Matrixmetalloproteinase-13
Nrf-2: Nuclear factor erythroid 2-related factor 2
PLK1: Polo-like kinase 1
RUNX2: Runt-related transcription factor 2
siRNA: Small interfering RNA
shRNA: Small hairpin RNA
TA: Transactivation
TD1: Transactivation domain I
VEGF: Vascular endothelial growth factor
VHL: Von Hippel-Lindau
